# Pharmacological targeting of xCT/system Xc− to modulate temozolomide sensitivity in glioma and glioblastoma preclinical models: a focused systematic review

**DOI:** 10.1007/s00280-026-04957-y

**Published:** 2026-10-09

**Authors:** Sufyan Elhashani, Mohamed Elkawafi, Abdalmanam El Shekhi, Sanad Elrishe, Mahmoud A. Aloriby

**Affiliations:** 1 Division of Medical Education, School of Medical Sciences, University of Manchester, Manchester, UK; 2 Basic Medical Sciences Program, School of Health and Medical Sciences, Libyan International University, Benghazi, Libya; 3 Department of Neurology, Lancashire Teaching Hospitals NHS Foundation Trust, Preston, UK; 4 Department of Critical Care, Lancashire Teaching Hospitals NHS Foundation Trust, Preston, UK; 5 Department of Pathology, Medical Center, Libyan International Medical University, Benghazi, Libya

**Keywords:** Glioblastoma, Glioma, Temozolomide, Chemotherapy resistance, xCT, SLC7A11, System Xc−, Sulfasalazine, Erastin, Sorafenib, Ferroptosis

## Abstract

**Background:**

Resistance to temozolomide (TMZ) remains a major barrier in glioblastoma treatment. The cystine/glutamate antiporter system Xc−, mainly through its xCT/SLC7A11 light-chain subunit, supports cystine uptake, glutathione synthesis and redox balance, making it a biologically plausible target for modifying TMZ response. This focused systematic review evaluated whether pharmacological xCT/system Xc−-relevant inhibition enhances TMZ sensitivity in glioma/glioblastoma preclinical models.

**Methods:**

Searches were conducted in Web of Science, PubMed/MEDLINE and the Cochrane Library, with supplementary Google Scholar screening, and were completed on 30 April 2026. Reference lists of the included preclinical studies were also screened as a supplementary citation-chasing method. No lower publication-date restriction was applied. Eligible studies were primary preclinical studies using glioma/glioblastoma models, TMZ treatment, and TMZ combined with a pharmacological xCT/system Xc−-relevant inhibitor or related intervention. Because the included studies differed in models, inhibitors, formulations, treatment conditions and outcome measures, findings were synthesised narratively.

**Results:**

Ten non-retracted preclinical studies were included. Free-drug Sulfasalazine/SSZ and erastin studies provided the most directly relevant pharmacological evidence, but their effects differed by cell model, concentration, treatment duration and outcome assay. Genetic SLC7A11 manipulation supported a relationship between xCT expression, redox regulation and TMZ response, but was not treated as equivalent to pharmacological inhibition. Recent TMZ/SSZ and TMZ/erastin delivery-platform studies produced antitumour effects in TMZ-resistant and orthotopic models; however, these findings also reflected altered circulation, BBB or tumour delivery, targeting and sustained release. Sorafenib/TMZ results were inconsistent and generally lacked direct xCT/SLC7A11 assessment.

**Conclusions:**

xCT/system Xc− targeting is a promising but preliminary preclinical strategy for modifying TMZ response. Evidence remains heterogeneous, model- and formulation-dependent, and insufficient to support clinical efficacy claims. Genetic SLC7A11 studies provide mechanistic support, while selected free-drug SSZ and erastin experiments provide limited pharmacological evidence; recent in-vivo findings are predominantly formulation-specific, and sorafenib evidence remains mixed and non-specific. Further target-engagement, pharmacokinetic, BBB, toxicity and orthotopic validation is required.

**Supplementary Information:**

The online version contains supplementary material available at https://doi.org/10.1007/s00280-026-04957-y.

## Background

Gliomas are brain tumours that arise from glial cells and related glial cell populations. Among these tumours, glioblastoma is considered the most aggressive and clinically challenging form. Despite advances in surgery, radiotherapy and chemotherapy, glioblastoma remains associated with poor prognosis and limited long-term survival. This is mainly due to invasive growth, molecular heterogeneity and the ability of glioma cells to acquire resistance to anticancer treatments [1, 2]. Temozolomide (TMZ) is an orally administered alkylating agent and remains an important chemotherapeutic drug in glioblastoma treatment. Its relatively small size and lipophilic nature allow it to penetrate the blood–brain barrier, making it more suitable than many other chemotherapeutic drugs for treating brain tumours. TMZ exerts its anticancer effect mainly through DNA methylation, especially the formation of O6-methylguanine, which can trigger DNA mismatch repair, cell cycle arrest and cell death [3, 4]. However, a substantial number of glioblastoma patients do not respond effectively to TMZ, and acquired TMZ resistance remains one of the main reasons for treatment failure [4].

Several mechanisms have been linked to TMZ resistance in glioma. The most recognised mechanism is upregulation of O6-methylguanine-DNA-methyltransferase (MGMT), which repairs TMZ-induced O6-methylguanine lesions. Alterations in base excision repair, mismatch repair, p53 function and cellular stress-response pathways have also been associated with TMZ resistance [5, 6]. However, these mechanisms do not fully explain why some glioma cells remain resistant to TMZ. Therefore, additional pathways that regulate TMZ sensitivity need to be assessed. One pathway of interest is intracellular redox regulation. TMZ-induced damage can be associated with increased reactive oxygen species (ROS), which contributes to cellular stress and cell death. However, glioma cells can counteract ROS accumulation through antioxidant systems, especially glutathione (GSH). GSH synthesis depends on intracellular cysteine availability, which makes cystine uptake and cysteine metabolism important regulators of cellular redox balance [7, 8]. In glioma cells, cystine uptake is largely facilitated by system Xc−, a cystine/glutamate antiporter composed of the xCT/SLC7A11 light-chain subunit and the 4F2hc/CD98 heavy-chain subunit [7, 9].

The upregulation of xCT/system Xc− has been associated with glioma progression, glutamate secretion, redox adaptation and chemoresistance. Increased xCT expression may allow glioma cells to tolerate treatment-induced oxidative stress by maintaining cysteine availability and GSH synthesis. Therefore, pharmacological inhibition of xCT/system Xc− may reduce GSH availability, increase ROS accumulation and increase TMZ cytotoxicity in selected models. Inhibition of system Xc− can also be consistent with ferroptosis-related vulnerability because reduced cystine uptake can impair GSH-dependent lipid peroxide detoxification. However, ferroptosis should not be assumed unless direct ferroptosis markers or rescue experiments are reported. Several pharmacological agents have been used as xCT/system Xc−-relevant inhibitors in glioma research, including sulfasalazine (SAS), erastin and sorafenib. These agents are not fully equivalent. SAS is widely discussed as an inhibitor of system Xc−, erastin is classically linked to system Xc− inhibition and ferroptosis induction, and sorafenib has multiple targets in addition to reported effects on cystine-glutamate exchange [10–12]. Therefore, these drugs need to be interpreted carefully when they are used in combination with TMZ. Because these interventions are pharmacological strategies intended to alter chemotherapy response, a focused synthesis of the preclinical evidence is useful for clarifying whether xCT/system Xc− targeting is a plausible TMZ-sensitising approach.

This focused systematic review aimed to evaluate whether pharmacological inhibition of xCT/system Xc− enhances TMZ sensitivity in glioma/glioblastoma preclinical models. Broader ferroptosis/TMZ papers were considered only as background unless they directly tested TMZ with an xCT/system Xc−-relevant pharmacological intervention in glioma models.

## Methods

### Review design and registration

This focused systematic review aimed to identify primary preclinical studies assessing TMZ cotreatment with pharmacological xCT/system Xc−-relevant inhibitors, or pharmacological interventions with reported relevance to xCT/system Xc− or cystine/GSH-dependent ferroptosis, in glioma/glioblastoma models. The review was not prospectively registered; therefore, no registration number is available.

### Search strategy

Searches were conducted between October 2025 and April 2026 and were completed on 30 April 2026. Records were identified from Web of Science, PubMed/MEDLINE and the Cochrane Library, with supplementary screening in Google Scholar. No lower publication-date restriction was applied. The search strategy combined terms for glioma/glioblastoma, temozolomide/TMZ, xCT/SLC7A11/system Xc−, cystine/glutamate antiporter, sulfasalazine, erastin, sorafenib, sensitivity/resistance, cytotoxicity, viability, ferroptosis, reactive oxygen species and glutathione. The strategy was adapted to the syntax of each source, and the database-specific search strings are presented in Supplementary Table 1. The first 60 Google Scholar results were screened manually because Google Scholar was used as a supplementary source rather than as a reproducible database export. Reference lists and relevant in-text citations were also checked, but the number of records identified through citation checking was not recorded and was therefore reported narratively rather than included in the numerical PRISMA count.

### Eligibility criteria

Studies were eligible if they were primary preclinical research studies; used glioma or glioblastoma models; included TMZ treatment; assessed TMZ in combination with a pharmacological inhibitor considered relevant to xCT/system Xc− inhibition, including SAS, erastin or sorafenib; and reported outcomes related to TMZ sensitivity, cell viability, cytotoxicity, apoptosis, ferroptosis-related changes, ROS, GSH, lipid peroxidation, GPX4, xCT/SLC7A11 expression, tumour growth or survival. Sorafenib studies were eligible only where TMZ cotreatment was experimentally assessed, and were interpreted as supportive or borderline multi-target evidence unless xCT/SLC7A11 or system Xc− function was directly evaluated. Genetic xCT/SLC7A11 overexpression or knockdown data were extracted as supportive mechanistic evidence, but were not treated as equivalent to pharmacological inhibition.

Studies were excluded if they were reviews, editorials, commentaries or protocols; did not use glioma/glioblastoma models; did not include TMZ; did not assess a pharmacological xCT/system Xc−-relevant inhibitor or closely related inhibitor in combination with TMZ; were clinical-only studies; or used broader ferroptosis interventions without clear relevance to xCT/system Xc− or the TMZ response question. Clinical studies, clinical trial protocols and ongoing clinical trials were excluded from the core synthesis because the review question focused on preclinical glioma/glioblastoma models. Relevant clinical or translational records identified during screening were retained only as background records where they helped contextualise clinical translation, tolerability or future research [24–26]. Retracted articles were not used as reliable evidence in the final synthesis.

### Study selection and data extraction

Records from all sources were collated into a master screening list containing title, author, publication year, journal, DOI and abstract or search-result information where available. Duplicates were identified manually using DOI, exact title and combinations of author, year and journal information. Complete automated export and duplicate-removal records were not available for every source; consequently, the final source and duplicate counts were reconstructed from saved search outputs and manual screening records. This process and its limitations were recorded in the review documentation. All five authors independently searched the selected databases using predefined search terms and eligibility criteria. Titles and abstracts were screened against the eligibility criteria, and full texts were assessed where eligibility could not be determined from the title and abstract alone. Potentially eligible studies identified by any author were discussed by the full author team. Disagreements or uncertainties were resolved through group discussion, and the final list of included studies was agreed by consensus.

Data were extracted on publication details, cell models, TMZ and inhibitor concentrations, treatment duration, comparators, cell viability, cytotoxicity, apoptosis/cell death, ROS, GSH, lipid peroxidation, GPX4, xCT/SLC7A11 expression, gene expression, metabolic findings, tumour growth, survival and study limitations. Screening and data extraction were performed by the review team. Eligibility decisions, extracted data and study classifications were checked by the authors during manuscript preparation to improve consistency.

### Quality assessment and synthesis

Study quality and risk-of-bias were assessed using a structured checklist developed for this review and informed by preclinical, in-vitro and animal-study reporting domains [27, 28]. The assessment covered model identification and relevance, reporting of drug concentrations and treatment duration, comparator and single-agent groups, outcome measurement, statistical analysis, reproducibility, randomisation and blinding where applicable, direct assessment of xCT/system Xc−, ferroptosis validation, in-vivo relevance and acknowledgement of study limitations. Items were recorded as yes, no, not applicable or unclear, with support-for-judgement notes.

Findings are presented as a study-level summary in Supplementary Table 4. A composite numerical score was not calculated because the importance of individual domains differed according to the study question and design. Instead, the assessment was used to guide interpretation. Studies with direct xCT/SLC7A11 manipulation or functional assessment were given greater weight for mechanistic conclusions; studies without direct target assessment were classified as indirect or multi-target evidence. Delivery-platform studies were interpreted as evidence for the complete formulation rather than as proof of equivalent free-drug activity. Findings based mainly on metabolic viability assays, high or clinically uncertain concentrations, limited models, incomplete ferroptosis validation or incompletely reported randomisation and blinding were interpreted cautiously.

Meta-analysis was not performed because the included studies differed substantially in cell and animal models, inhibitors, concentrations, treatment durations, formulations, routes of administration and outcome measures. Findings were synthesised narratively by inhibitor type, evidence format and directness of xCT/system Xc− assessment.

## Results

### Study selection

**Fig. 1:**
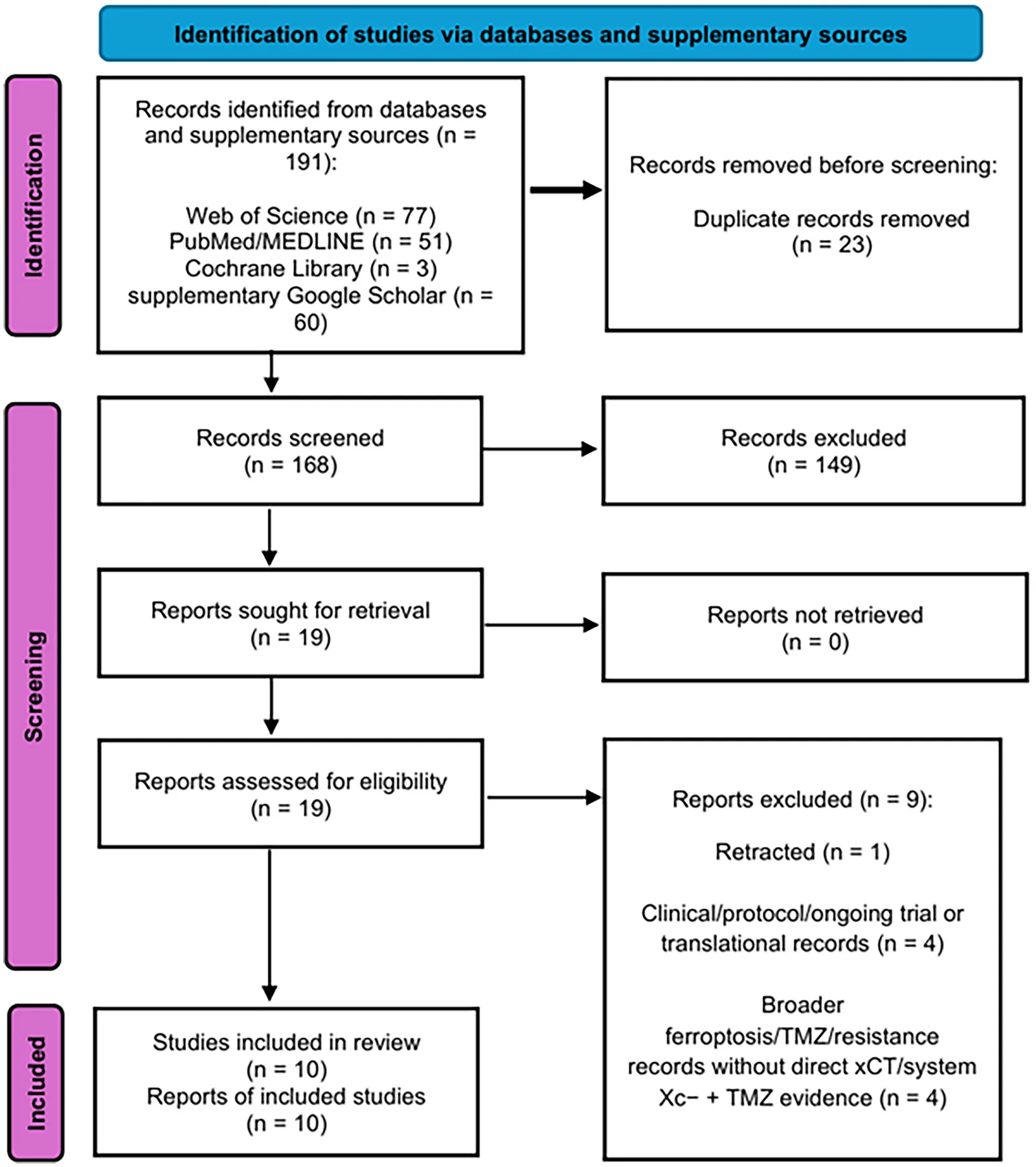
PRISMA 2020 flow diagram for study selection. Searches were conducted between October 2025 and April 2026 and completed on 30 April 2026. Records were identified from Web of Science, PubMed/MEDLINE and the Cochrane Library, with supplementary Google Scholar screening. Duplicate records were removed manually using DOI, title, author, year and journal. Ten non-retracted preclinical studies were included in the final narrative synthesis

The study selection process is summarised in Fig. 1. Searches identified 191 records before duplicate removal: 77 from Web of Science, 51 from PubMed/MEDLINE, 3 from the Cochrane Library and 60 from supplementary Google Scholar screening. Citation and reference checking was also performed, but the number of records identified through this route was not recorded and was therefore not included in the numerical PRISMA count. After manual duplicate removal using DOI, title, author, publication year and journal information, 23 duplicate records were removed, leaving 168 unique records for title and abstract screening. Of these, 149 were excluded because they did not meet the review question or were reviews, clinical or protocol records, broader ferroptosis/TMZ studies, non-glioma studies, studies without TMZ or studies without pharmacological xCT/system Xc−-relevant targeting. Nineteen reports were assessed in detail. Nine reports were excluded after detailed assessment: one was retracted, four were clinical/protocol/ongoing trial or translational records [13], and four addressed broader ferroptosis/TMZ/resistance mechanisms without directly testing pharmacological xCT/system Xc− inhibition with TMZ in glioma/glioblastoma preclinical models. The four clinical, protocol or translational exclusions comprised three sulfasalazine-related records [24–26] and one sorafenib-based record [30]. Their individual exclusion reasons are provided in Supplementary Table 3. Ten non-retracted preclinical studies were included in the final narrative synthesis [14–23]. The ten included studies were Ignarro et al. 2016, Sehm et al. 2016a, Polewski et al. 2016, Sehm et al. 2016b, Wei et al. 2021, Riedel et al. 2016, Jakubowicz-Gil et al. 2017, Zhang et al. 2023, Wang et al. 2023 and Yi et al. 2025 [14–23]. Their relevance to the review question differed: SAS/SSZ and erastin studies provided the most directly relevant pharmacological evidence, whereas most sorafenib studies were classified as supportive or borderline because xCT/SLC7A11 was not directly assessed.

### Quality and risk-of-bias considerations

The included studies varied in methodological directness and translational relevance. Polewski et al. and Sehm et al. 2016a provided the strongest mechanistic evidence because xCT/SLC7A11 was experimentally manipulated and linked to TMZ response. Sehm et al. 2016b also assessed xCT-related activity and used ferroptosis-rescue experiments, but its direct TMZ-combination evidence was limited and its in-vivo SAS treatment did not clearly reduce tumour growth or improve survival. Ignarro et al. directly tested free TMZ and SAS but did not measure xCT/system Xc− activity, so it was treated as indirect pharmacological evidence. The sorafenib studies were assigned lower confidence for xCT-specific interpretation because most did not assess xCT/SLC7A11. Riedel et al. nevertheless provided important negative evidence through clonogenic survival across six GBM lines, whereas other sorafenib studies relied mainly on shorter-term viability, apoptosis or formulation-based outcomes. Wang et al. and Yi et al. included TMZ-resistant and in-vivo models with direct xCT-related measurements, but their findings were interpreted as platform-specific because targeting, altered exposure and sustained release contributed to the observed effects.

Common limitations included reliance on established cell lines, wide variation in drug concentrations and treatment schedules, use of metabolic assays as proxies for viable cell number, inconsistent ferroptosis rescue, small animal groups and incomplete reporting of blinding. These considerations limited direct comparison and reduced confidence in broad or clinically predictive conclusions (Table 1).

**Table 1:** Summary of included studies and interpretive limitations

Study [references]	Key model(s)	Intervention/exposure	Evidence format and xCT/system Xc− directness	Main relevant findings	Key interpretive limitation
Ignarro et al. 2016 [15]	A172 and T98G human GBM cells	25 µM TMZ ± 0.5 mM SAS for 1, 3 or 5 days	Free drugs; indirect xCT-relevant evidence because xCT/system Xc− was not directly measured	TMZ alone did not alter viability; TMZ+SAS reduced viability after 5 days in both lines. Synergy was reported in A172 at 3 and 5 days. SAS depleted total GSH in A172	In vitro; one high SAS concentration; MTT-based viability; no direct target-engagement or ferroptosis-rescue experiment
Sehm et al. 2016a [16]	F98 and U251 xCT-overexpressing/knockdown glioma cells	TMZ with SAS, erastin or sorafenib	Free drugs plus genetic xCT manipulation; direct/partly direct mechanistic evidence	TMZ response varied with xCT expression, particularly in U251. Erastin-induced death was rescued by DFO/Fer-1; erastin/TMZ was additive in selected conditions. TMZ-induced death itself was not rescued. Sorafenib/TMZ effects were inconsistent	Condition- and model-specific findings; high TMZ exposure in the rescue experiment; no in vivo validation
Polewski et al. 2016 [17]	U251, U87, pNHA, PBT017; U251 SLC7A11 overexpression/knockdown	TMZ+SSZ dose matrix; genetic SLC7A11 manipulation	Free drugs plus genetic manipulation; direct mechanistic evidence with a pharmacological component	Low SSZ concentrations showed synergy with TMZ in U251. SLC7A11 overexpression increased TMZ resistance and altered redox, mitochondrial and metabolic features	TMZ-response experiments centred mainly on U251; in vitro; genetic manipulation is not equivalent to pharmacological inhibition
Sehm et al. 2016b [18]	F98, U251, astrocytes/neurons, ex vivo and orthotopic SAS models	TMZ+SAS in vitro; SAS alone in ex vivo and in vivo microenvironment models	Free-drug SAS/xCT evidence; direct xCT/ferroptosis assessment, but limited TMZ-combination evidence	High-concentration SAS-induced death was rescued by DFO/Fer-1. TMZ+SAS was additive in U251 but not F98. In vivo SAS reduced oedema but did not clearly reduce tumour growth or improve survival	Cytotoxicity occurred mainly at high SAS concentrations; small animal groups; in vivo treatment did not test TMZ+SAS
Wang et al. 2023 [22]	U251TR, LN229TR and GL261TR TMZ-resistant GBM models; postoperative resection model	Free TMZ:erastin ratio 40:1, followed by cRGD-liposome/GelMA co-delivery	Free-drug interaction followed by a local targeted delivery platform; direct xCT and ferroptosis-related measurements	Free TMZ+erastin showed combination indices of 0.67–0.73. The platform reduced xCT, GPX4, ferritin, MGMT and GSH, increased ROS/MDA and inhibited postoperative recurrence; median survival reached 76.5 days	The in vivo effect combines drug interaction with local implantation, targeting and sustained release; no ferroptosis-rescue experiment for the combination
Yi et al. 2025 [23]	GL261/TR cells, bEnd.3 BBB model and orthotopic GL261/TR tumours	Free TMZ+SSZ and PCB-modified co-loaded liposomes; in vivo TMZ 44 mg/kg and SSZ 17.6 mg/kg	Systemic targeted liposomal platform; direct xCT and redox measurements	PCB liposomes prolonged circulation, improved BBB/tumour delivery, suppressed xCT, depleted GSH/GPX4, increased ROS/LPO and Fe2+, reduced MGMT, inhibited tumour growth and prolonged median survival to 36.5 days	Platform-specific effect; murine resistant model; high in vitro concentrations; ferroptosis was not confirmed by a rescue experiment
Wei et al. 2021 [14]	U251, SHG-44, C6 and LN18; subcutaneous U251 xenografts	2 µM sorafenib+100 µM TMZ in vitro; 20 mg/kg sorafenib+25 mg/kg TMZ in vivo	Free drugs; indirect multi-target evidence because xCT/SLC7A11 was not assessed	The combination reduced viability, increased apoptosis and inhibited xenograft growth through ROS, autophagy and JAK2/STAT3-AIF signalling	No direct xCT evidence; subcutaneous model; short-term metabolic outcomes; small animal groups and no BBB exposure measurement
Riedel et al. 2016 [19]	Six MGMT-negative GBM cell lines	Sorafenib 5 µg/ml with TMZ 1–10 µM; clonogenic survival	Free drugs; negative indirect multi-target evidence	Sorafenib inhibited proliferation but failed to enhance TMZ-induced long-term cell inactivation and produced resistance-like effects in some lines	In vitro; no xCT/ferroptosis assessment; one principal sorafenib exposure; all lines were MGMT-negative
Jakubowicz-Gil et al. 2017 [20]	T98G GBM and MOGGCCM anaplastic astrocytoma cells	Sorafenib and TMZ alone or together for 48 h	Free drugs; indirect programmed-cell-death evidence	The combination promoted cell-line-specific programmed cell death, mainly apoptosis, with ER stress preceding elimination	Very high printed drug concentrations; no xCT assessment or in vivo validation; microscopy and flow-cytometry estimates differed substantially
Zhang et al. 2023 [21]	U87MG cells and U87MG tumour-bearing mice	LAT1-targeted nanoparticles co-loaded with TMZ and sorafenib	Targeted nanoparticle platform; no direct xCT assessment	LAT1-targeted co-delivery increased uptake, apoptosis, ROS, GSH depletion, GPX4 reduction and antitumour activity compared with non-targeted nanoparticles	Effects combine drug activity with LAT1 targeting and altered delivery; U87MG-dominant model; no ferroptosis-rescue experiment

BBB, blood–brain barrier; DFO, desferoxamine; Fer-1, ferrostatin-1; GBM, glioblastoma; GSH, glutathione; LAT1, L-type amino acid transporter 1; LPO, lipid peroxidation; MDA, malondialdehyde; MGMT, O6-methylguanine-DNA-methyltransferase; PCB, poly(carboxybetaine); SAS/SSZ, sulfasalazine; TMZ, temozolomide.

### Sulfasalazine and TMZ cotreatment

SAS/SSZ was the most directly relevant xCT/system Xc− inhibitor in the non-retracted evidence base. Ignarro et al. reported that 25 µM TMZ alone did not significantly alter A172 or T98G viability, whereas 25 µM TMZ combined with 0.5 mM SAS reduced viability after 5 days in both cell lines. The drug combination was described as synergistic in A172 cells after 3 and 5 days, but not in T98G cells [15]. In A172 cells, SAS alone and SAS/TMZ also markedly depleted total glutathione, while TMZ alone did not significantly alter total glutathione [15]. This supports a redox-based interpretation, although xCT activity itself was not directly measured in that paper.

Polewski et al. reported that parental U251 cells treated with increasing TMZ and SSZ concentrations showed synergy at 50 µM SSZ across TMZ doses and at other lower dose combinations, whereas high SSZ concentrations were additive or antagonistic [17]. Sehm et al. 2016a also reported that TMZ and SAS responses were influenced by xCT expression, particularly in xCT knockdown glioma cells [16]. Sehm et al. 2016b found no clear additive TMZ/SAS effect in F98 cells but reported an additive effect in U251 cells [18]. Collectively, the SAS evidence supports a possible TMZ-sensitising effect in selected glioma models, but the effect is not uniform across cell lines or concentrations.

Yi et al. 2025 further strengthened the SAS/TMZ evidence by evaluating a poly(carboxybetaine)-modified liposomal system co-delivering TMZ and sulfasalazine in GL261/TR TMZ-resistant glioma cells and an orthotopic TMZ-resistant GBM mouse model [23]. In vitro, the study compared TMZ, SSZ, free TMZ+SSZ, non-targeted liposomal formulations and T&S@LPS-PCB. T&S@LPS-PCB produced the strongest suppression of GL261/TR viability, colony formation, migration and invasion, and increased apoptosis-related changes. Mechanistically, the formulation suppressed x-CT expression, reduced GSH and GPX4, increased ROS and lipid peroxidation, increased Fe2+ accumulation through DMT1 upregulation, and reduced β-catenin and MGMT expression [23]. In vivo, T&S@LPS-PCB improved blood–brain barrier (BBB) penetration and tumour targeting, reduced orthotopic TMZ-resistant GBM growth and prolonged median survival compared with free TMZ, free SSZ, TMZ+SSZ and other liposomal controls [23]. This study provides formulation-specific evidence for a TMZ/SSZ strategy associated with xCT and redox changes. However, it does not establish that free SSZ/TMZ would reproduce the same intracranial exposure or antitumour effect**.**

### Erastin and TMZ cotreatment

After removal of the retracted Chen et al. study [13], the non-retracted erastin/TMZ evidence is no longer limited to Sehm et al. 2016a. Sehm et al. 2016a tested erastin with TMZ in xCT-modulated glioma cells and reported additive cytotoxic effects in some conditions [16]. In Sehm et al. 2016a, erastin-induced cell death was rescued by deferoxamine and ferrostatin-1, supporting ferroptosis involvement. However, TMZ-induced cell death itself was not rescued by these agents, indicating that TMZ alone did not induce ferroptosis in that rescue assay. Therefore, this study supports erastin-based ferroptosis potentiation of TMZ rather than TMZ alone acting through ferroptosis. Wang et al. 2023 substantially strengthened the erastin/TMZ evidence by using a TMZ-resistant GBM model and a hydrogel-liposome system co-delivering TMZ and erastin [22]. In that study, TMZ-resistant U251TR and LN229TR cells were established by repetitive TMZ exposure, free TMZ and erastin showed synergistic cytotoxicity at a 40:1 ratio, and the T+E@LPs-cRGD+GelMA platform reduced proliferation, viability, migration and invasion in TMZ-resistant GBM cells [22].

Mechanistically, Wang et al. reported reduced xCT, GPX4 and ferritin expression, decreased GSH, increased MDA, ROS accumulation and ferroptosis-like mitochondrial changes following TMZ/erastin delivery [22]. MGMT expression was also reduced and p53 increased. These findings support the biological plausibility that erastin-mediated xCT/ferroptosis targeting can contribute to TMZ sensitivity in resistant GBM models. However, this evidence comes from a complex delivery platform, so the effects reflect both drug combination and targeted/sustained delivery rather than erastin alone.

### Sorafenib and TMZ cotreatment

Sorafenib evidence requires careful interpretation because sorafenib has multiple pharmacological targets and was not consistently tested as an xCT/system Xc− inhibitor. Sehm et al. 2016a assessed sorafenib with TMZ in xCT-modulated F98 and U251 cells, but the sorafenib/TMZ effects were not consistently additive, especially in human U251 cells [16]. Wei et al. 2021 reported that sorafenib enhanced TMZ cytotoxicity in U251 and SHG-44 glioma cells and inhibited tumour growth in a U251 xenograft model [14]. However, Wei et al. linked the combination to oxidative stress, autophagy and JAK2/STAT3-AIF signalling rather than direct xCT/system Xc− inhibition [14].

Across the full-text studies, sorafenib/TMZ findings were mixed. Jakubowicz-Gil et al. reported that TMZ and sorafenib together promoted programmed cell death in T98G and MOGGCCM cells, with apoptosis dominating after simultaneous drug application and ER stress preceding cell elimination [20]. Zhang et al. reported that LAT1-targeted nanoparticles co-loaded with TMZ and sorafenib increased glioma-cell uptake, cytotoxicity, apoptosis, ROS, GSH depletion, GPX4 reduction and antitumour efficacy in a U87MG model [21]. In contrast, Riedel et al. used clonogenic assays across six MGMT-negative GBM cell lines and found that sorafenib failed to enhance TMZ-induced cell killing, with resistance-like effects in some settings [19]. Therefore, sorafenib should be treated as supportive and heterogeneous multi-target evidence, not direct xCT-specific evidence.

### Supportive genetic and mechanistic xCT/SLC7A11 evidence

Genetic xCT/SLC7A11 manipulation provided supportive evidence that xCT expression can influence TMZ response. Polewski et al. showed that SLC7A11 overexpression increased TMZ resistance, whereas SLC7A11 knockdown increased TMZ sensitivity and altered ROS/GSH balance [17]. The same paper also showed that SLC7A11 overexpression was associated with altered cellular metabolism, increased mitochondrial biogenesis, oxidative phosphorylation and ATP generation [17]. These findings strengthen the biological rationale that xCT/SLC7A11 can influence TMZ response, but genetic manipulation should not be treated as identical to pharmacological inhibition.

### Redox and ferroptosis-related outcomes

The evidence base supports redox and ferroptosis-related mechanisms in selected studies. Several studies measured GSH, ROS, lipid peroxidation, GPX4 or ferroptosis-rescue outcomes. Sehm et al. 2016b used DFO and ferrostatin-1 rescue to support SAS-induced ferroptosis at high SAS concentrations [18]. Wang et al. 2023 measured xCT, GPX4, ferritin, GSH, MDA, ROS and mitochondrial changes in TMZ-resistant GBM models after TMZ/erastin delivery [22]. Yi et al. 2025 measured x-CT, GSH, GPX4, ROS, lipid peroxidation, DMT1, Fe2+ accumulation, β-catenin and MGMT after TMZ/SSZ liposomal delivery in TMZ-resistant GBM models [23]. Zhang et al. 2023 measured ROS, GSH and GPX4 changes after TMZ/sorafenib nanoparticle delivery [21]. Nevertheless, direct ferroptosis confirmation was not consistent across all TMZ combination experiments, and most sorafenib studies did not test xCT/SLC7A11. The mechanism should therefore be described as redox-based and ferroptosis-compatible, with stronger ferroptosis evidence in selected studies rather than across the whole evidence base.

## Discussion

This review identifies a preclinical evidence base that is biologically coherent but methodologically heterogeneous. The most directly relevant pharmacological evidence comes from SAS/SSZ and erastin, while genetic SLC7A11 studies provide mechanistic support and most sorafenib studies remain indirect because of the drug's multi-target pharmacology. The central interpretive question is therefore not whether all xCT/system Xc−-relevant interventions improve TMZ response, but under which models, exposures and delivery conditions a sensitising effect is observed.

Across the free-drug studies, SAS/SSZ effects were model- and concentration-dependent: positive interactions were reported in selected A172 and U251 experiments, whereas effects were weaker or absent in other models including T98G and F98. Erastin also produced additive or synergistic interactions in selected settings, but the strongest recent in-vivo evidence for both SSZ and erastin comes from specialised delivery systems. The studies by Yi et al. and Wang et al. therefore extend the preclinical evidence into resistant and orthotopic models without establishing that the corresponding free drugs would achieve equivalent intracranial exposure or efficacy. Sorafenib provides less direct support for the xCT/TMZ hypothesis. Positive short-term or formulation-based findings in Wei et al., Jakubowicz-Gil et al. and Zhang et al. are counterbalanced by the negative clonogenic findings of Riedel et al.; the overall evidence is therefore neither consistently sensitising nor xCT-specific. Across inhibitor classes, disruption of GSH and ROS regulation and other ferroptosis-compatible changes recur, but direct ferroptosis validation was inconsistent and apoptosis or autophagy also contributed in several models.

The experimental concentration ranges limit direct pharmacological comparison. Ignarro et al. combined 25 µM TMZ with 0.5 mM SAS and reported synergy in A172 but not T98G cells. Polewski et al. observed synergy at 50 µM SSZ across the tested TMZ range, whereas combinations containing substantially higher SSZ concentrations were more likely to be additive or antagonistic. Sehm et al. 2016b reported little SAS cytotoxicity below approximately 200 µM and ferroptotic cell death mainly at higher concentrations. These findings indicate that the interaction is not explained by inhibitor identity alone and depends on whether the concentration produces selective system Xc− interference, broader cellular stress or direct toxicity. The sorafenib studies also differed in pharmacological conditions and outcome selection. Wei et al. reported enhanced short-term cytotoxicity using 2 µM sorafenib with 100 µM TMZ, whereas Riedel et al. used 1–10 µM TMZ with approximately 7.8 µM sorafenib and found no improvement in long-term clonogenic survival. Jakubowicz-Gil et al. reported very high drug concentrations, which substantially limits clinical interpretation. Differences between metabolic viability, apoptosis and clonogenic assays may therefore contribute to the conflicting sorafenib results. In particular, short-term reduction in metabolic activity or increased apoptosis does not necessarily demonstrate durable loss of tumour-cell reproductive capacity.

BBB exposure and route of administration further separate free-drug from delivery-platform evidence. Free-drug studies test pharmacological interactions under defined experimental exposure, whereas platform studies combine drug effects with carrier properties, route, targeting and release kinetics. Wang et al. used a locally implanted GelMA hydrogel containing cRGD-targeted TMZ/erastin liposomes, reducing dependence on systemic BBB penetration; Yi et al. used PCB-modified liposomes to prolong circulation and increase BBB transport and tumour accumulation; and Zhang et al. used LAT1-targeted nanoparticles to improve drug delivery. These studies therefore provide formulation-specific evidence and cannot be taken as direct confirmation that free erastin, SSZ or sorafenib would reproduce the same intracranial exposure or antitumour effects.

The available studies also do not establish that the active free-drug concentrations can be achieved safely within human GBM tissue, particularly for SAS/SSZ, for which high in-vitro concentrations were often required. Clinical and protocol records excluded from the core synthesis therefore provide useful, although indirect, context for translational feasibility. Robe et al. described a phase 1–2 clinical protocol of sulfasalazine in progressing malignant glioma, while the GLUGLIO trial is evaluating gabapentin, sulfasalazine and memantine as add-on treatment to standard chemoradiotherapy with TMZ in newly diagnosed GBM [24, 25]. Takeuchi et al. provides useful caution because sulfasalazine with TMZ and radiotherapy did not show a clear improvement in overall or progression-free survival and was associated with substantial grade 3/4 toxicity in a small clinical cohort [26]. Similar caution applies to sorafenib-based clinical combinations, as a phase I study of sorafenib with radiotherapy and TMZ in high-grade glioma reported significant side effects and only moderate outcome results and did not support further clinical development of that regimen [30]. Therefore, promising preclinical xCT/system Xc−-relevant findings should be interpreted alongside dosing feasibility, blood–brain barrier delivery and potential added toxicity before clinical translation is considered. These clinical records support translational interest in glutamate/system Xc−-related pathways, but also reinforce that preclinical TMZ/xCT findings should not be interpreted as evidence of clinical efficacy.

Future studies should use patient-derived glioblastoma cells, glioma stem-like cells, orthotopic models and standardised TMZ/inhibitor concentrations. They should measure xCT/SLC7A11 expression, cystine uptake, extracellular glutamate, GSH, ROS, lipid peroxidation, GPX4 activity and ferroptosis rescue with agents such as ferrostatin-1 or liproxstatin-1. Studies should also compare free-drug combinations with delivery-platform effects so that pharmacological sensitisation can be separated from improved drug delivery. Future studies should also better align preclinical xCT/system Xc− inhibitor experiments with clinically feasible treatment regimens, delivery approaches and safety considerations.

### Strengths and limitations

A strength of this review is its focused distinction between direct or partly direct xCT/system Xc− evidence, supportive genetic evidence, broader ferroptosis-associated findings, multi-target sorafenib evidence and delivery-platform studies. The synthesis also excluded retracted evidence and retained negative findings, including the failure of sorafenib to enhance clonogenic TMZ response in the study by Riedel et al. This reduces the risk of presenting the evidence as uniformly positive.

The underlying evidence base has important limitations. Most studies used established glioma cell lines, and patient-derived, stem-like and clinically representative TMZ-resistant models were limited. Models, concentrations, exposure durations, comparators and outcome assays varied substantially. Metabolic assays such as MTT and CCK-8 were common and may reflect altered metabolic activity as well as viable cell number. Direct xCT/SLC7A11 measurement or manipulation was absent from several included studies, particularly most sorafenib studies. Ferroptosis was not consistently confirmed through rescue experiments, and apoptosis or autophagy also contributed to treatment responses. The in-vivo evidence was heterogeneous. Some studies used subcutaneous xenografts, whereas others used orthotopic or postoperative models. The strongest recent antitumour findings involved nanoparticles, liposomes or hydrogels, making it difficult to separate pharmacological interaction from altered biodistribution, targeting or sustained release. Animal group sizes were generally limited, and blinding was frequently unreported. These factors restrict the extent to which findings can be compared or extrapolated to patients.

Only ten studies met the eligibility criteria, and the directly relevant pharmacological evidence was smaller than this overall count because several studies provided genetic mechanistic, indirect multi-target or formulation-specific evidence rather than direct free-drug xCT/system Xc− inhibition. Most studies did not establish unbound systemic or intracranial drug exposure, pharmacokinetic–pharmacodynamic relationships or functional xCT target engagement at the tested concentrations. High or clinically uncertain in-vitro exposures, off-target drug effects and the absence of human brain-tumour concentration data therefore limit translational interpretation. For liposome, nanoparticle and hydrogel studies, efficacy must also be interpreted alongside carrier safety, stability, release reproducibility, route feasibility and scalability, which were not established clinically.

The review process also has limitations. The review was not prospectively registered, and no formal certainty-grading system was applied. Google Scholar was used only as a supplementary source, and citation checking was not recorded numerically. Complete automated exports and duplicate-removal records were not available for every source; consequently, some source and screening counts were reconstructed from saved search outputs and manual records. This process has been described transparently, but it remains a limitation of reproducibility. The focused eligibility criteria also excluded broader GPX4-directed or ferroptosis-related TMZ studies that did not test an xCT/system Xc−-relevant pharmacological intervention [29]. These limitations mean that the review identifies biological plausibility and experimental priorities rather than estimating a pooled treatment effect or supporting a clinically ready combination.

## Conclusions

xCT/system Xc− targeting remains a promising but preliminary preclinical strategy for modifying TMZ response. Genetic SLC7A11 manipulation provides mechanistic support for a relationship between xCT status and TMZ response, whereas selected free-drug SSZ and erastin experiments provide limited, model-dependent pharmacological evidence. The overall evidence is heterogeneous and varies with model, concentration, treatment duration, assay and formulation. Delivery-platform studies provide preliminary formulation-specific evidence in resistant and orthotopic models, but do not establish equivalent free-drug efficacy or clinical benefit. Sorafenib/TMZ findings are mixed and largely non-specific to xCT/system Xc−. Further studies require clinically justified exposures, direct functional target-engagement measures, appropriate ferroptosis validation, patient-derived and TMZ-resistant models, orthotopic confirmation and pharmacokinetic, BBB and toxicity assessment before additional clinical efficacy evaluation can be justified.

## Supplementary Information

Below is the link to the electronic supplementary material.

Supplementary file1 (PDF 238 KB)

Supplementary file2 (PDF 234 KB)

Supplementary file3 (PDF 281 KB)

Supplementary file4 (PDF 289 KB)

Supplementary file5 (PDF 340 KB)

## Data Availability

No datasets were generated or analysed during the current study.

## Abbreviations

AIF: Apoptosis-inducing factor
BBB: Blood–brain barrier
BGT-1: Betaine/GABA transporter-1
CCK-8: Cell Counting Kit-8
CD98: Cluster of differentiation 98
CI: Combination index
cRGD: Cyclic arginine–glycine–aspartic acid peptide
DFO: Desferoxamine
DMT1: Divalent metal transporter 1
Fe2+: Ferrous iron
Fer-1: Ferrostatin-1
F98: Rat glioma cell line
GBM: Glioblastoma
GelMA: Gelatin methacryloyl
GL261/TR: Temozolomide-resistant GL261 glioma cells
GPX4: Glutathione peroxidase 4
GSH: Glutathione
LAT1: L-type amino acid transporter 1
LN18: Human glioblastoma cell line
LN229: Human glioblastoma cell line
LN229TR: Temozolomide-resistant LN229 glioma cells
LPO: Lipid peroxidation
MDA: Malondialdehyde
MGMT: O6-methylguanine-DNA-methyltransferase
MOGGCCM: Human anaplastic astrocytoma cell line
MRI: Magnetic resonance imaging
PCB: Poly(carboxybetaine)
PCNA: Proliferating cell nuclear antigen
p53: Tumour protein p53
pNHA: Primary normal human astrocytes
ROS: Reactive oxygen species
SAS: Sulfasalazine
SLC7A11: Solute carrier family 7 member 11
SSZ: Sulfasalazine
T98G: Human glioblastoma cell line
TMZ: Temozolomide
U87MG: Human glioblastoma cell line
U251: Human glioma cell line
U251TR: Temozolomide-resistant U251 glioma cells
xCT: Light-chain subunit of system Xc−
system Xc−: Cystine/glutamate antiporter

## Declarations

### Generative AI

During the preparation of this manuscript, the authors used an AI-assisted tool to support language editing and organisation of the manuscript. The authors reviewed and edited the content after using this tool and take full responsibility for the accuracy, integrity and final content of the manuscript.
