# Supplementary material for: Pharmacological targeting of xCT/system Xc− to modulate temozolomide sensitivity in glioma and glioblastoma preclinical models: a focused systematic review
Source: Cancer Chemother Pharmacol. 2026 Oct 9;96(1):116. doi: 10.1007/s00280-026-04957-y (PMC13652864; doi:10.1007/s00280-026-04957-y)
Supplement: Supplementary file 1 [file 280_2026_4957_MOESM1_ESM.pdf]

## Supplementary Table 1. Source-specific search strategies

| Source and platform/interface | Date searched | Exact strategy as executed | Fields searched | Filters/limits | Records retrieved |
|---|---|---|---|---|---|
| PubMed / MEDLINE | Oct 2025 - April 2026 | (glioma OR glioblastoma OR GBM OR astrocytoma) AND (temozolomide OR TMZ OR Temodal OR Temodar) AND (xCT OR SLC7A11 OR "system Xc" OR "system xc-" OR "cystine/glutamate antiporter" OR "cystine glutamate antiporter" OR sulfasalazine OR SAS OR SASP OR erastin OR sorafenib) AND (sensit* OR resistance OR cytotoxicity OR viability OR ferroptosis OR ROS OR glutathione OR GSH) | All fields | No lower publication-date restriction; no language restriction intentionally applied | 51 |
| Web of Science | Oct 2025 - April 2026 | (glioma OR glioblastoma OR GBM OR astrocytoma) AND (temozolomide OR TMZ) AND (xCT OR SLC7A11 OR "system Xc" OR "system xc-" OR "cystine/glutamate antiporter" OR sulfasalazine OR erastin OR sorafenib) AND (sensitivity OR resistance OR cytotoxicity OR viability OR ferroptosis OR ROS OR GSH) | All fields | No lower publication-date restriction; no language restriction intentionally applied | 77 |
| Cochrane Library | Oct 2025 - April 2026 | (glioma OR glioblastoma) AND (temozolomide OR TMZ) AND (sulfasalazine OR erastin OR sorafenib OR xCT OR SLC7A11) | All fields | No lower publication-date restriction; no language restriction intentionally applied | 3 |
| Google Scholar — web interface (supplementary search) | Oct 2025 - April 2026 | temozolomide glioma xCT SLC7A11 sulfasalazine erastin sorafenib; temozolomide glioma ferroptosis SLC7A11 | Google Scholar relevance-ranked results | First 60 results screened in order of relevance; no lower publication-date restriction. | 60 screened |
| Citation and reference-list checking | Oct 2025 - April 2026 | Manual checking of reference lists and relevant in-text citations from included and potentially eligible studies | Not applicable | Not applicable | Not recorded |

*Abbreviations: GBM, glioblastoma; GSH, glutathione; ROS, reactive oxygen species; TMZ, temozolomide.*
