# Supplementary material for: Pharmacological targeting of xCT/system Xc− to modulate temozolomide sensitivity in glioma and glioblastoma preclinical models: a focused systematic review
Source: Cancer Chemother Pharmacol. 2026 Oct 9;96(1):116. doi: 10.1007/s00280-026-04957-y (PMC13652864; doi:10.1007/s00280-026-04957-y)
Supplement: Supplementary file 2 [file 280_2026_4957_MOESM2_ESM.pdf]

Supplementary Table 2. Full-text eligibility assessment of included studies

Study	Primary research?	Glioma/GBM model?	TMZ included?	Pharmacological xCT/system Xc−-relevant intervention?	xCT/SLC7A11 directly measured or manipulated?	Eligible as core evidence?	Reason
Ignarro et al. 2016	Yes	Yes: A172 and T98G human glioblastoma cells	Yes	Yes: sulfasalazine/SAS combined with TMZ	No	Core indirect pharmacological evidence	Direct TMZ+SAS testing in human GBM cells. TMZ alone did not alter viability, whereas TMZ+SAS reduced viability after 5 days; synergy was reported in A172 cells. SAS depleted total glutathione, but xCT/system Xc− activity was not directly measured, so this is indirect SAS/system Xc−-relevant evidence.
Sehm et al. 2016a	Yes	Yes: F98 rat glioma and U251 human glioma cells with xCT overexpression or knockdown	Yes	Yes: SAS, erastin and sorafenib tested alone and/or with TMZ	Yes: xCT expression was experimentally modified; extracellular glutamate release was assessed	Core direct/partly direct mechanistic evidence	Directly relevant to the review question because it tested TMZ in xCT-modulated glioma cells and assessed xCT-relevant inhibitors. Human U251 xCT knockdown cells showed increased vulnerability to TMZ and TMZ/SAS. Erastin/TMZ showed additive effects in selected conditions. Erastin-induced death was rescued by DFO/Fer-1, but TMZ-induced death itself was not rescued, so TMZ alone should not be described as ferroptotic in this study. Sorafenib/TMZ effects were less consistent.
Polewski et al. 2016	Yes	Yes: U251, U87, pNHA, PBT017 and U251 SLC7A11 overexpression/ knockdown models	Yes	Yes: sulfasalazine/SSZ combined with TMZ; genetic SLC7A11 manipulation also used	Yes: SLC7A11 expression/manipulation, glutamate release, GSH and ROS-related measures	Supportive mechanistic evidence with pharmacological component	Eligible because it directly addresses SLC7A11/system Xc− biology and TMZ resistance, and includes SSZ/TMZ testing. SLC7A11 overexpression increased TMZ resistance, while

Study	Primary research?	Glioma/GBM model?	TMZ included?	Pharmacological xCT/system Xc−-relevant intervention?	xCT/SLC7A11 directly measured or manipulated?	Eligible as core evidence?	Reason
							knockdown increased TMZ sensitivity and altered ROS/GSH balance. Genetic evidence should be treated as supportive mechanistic evidence, not as equivalent to pharmacological inhibition.
Sehm et al. 2016b	Yes	Yes: F98, U251, astrocytes/neurons, ex vivo and in vivo glioma models	Yes, but TMZ-combination component is limited	Yes: SAS	Yes: SAS/xCT-related activity and ferroptosis-related effects assessed	Supportive SAS/xCT/ferroptosis evidence; limited direct TMZ-combination evidence	Eligible but should be interpreted cautiously. The study provides important SAS/xCT/ferroptosis evidence, including ferroptosis rescue with DFO/Fer-1 and effects on extracellular glutamate/tumour microenvironment. However, the direct TMZ/SAS-combination component is more limited than the broader SAS/xCT and oedema findings. In vivo SAS reduced glioma-associated oedema but did not clearly reduce tumour growth or survival.
Wei et al. 2021	Yes	Yes: U251, SHG-44, C6 and LN18 glioma cells; U251 xenograft model	Yes	Sorafenib combined with TMZ	No	Borderline/supportive multi-target evidence	Direct sorafenib+TMZ testing in glioma models with in vitro and in vivo outcomes. Sorafenib enhanced TMZ cytotoxicity and tumour inhibition through oxidative stress, autophagy and JAK2/STAT3-AIF signalling. However, xCT/SLC7A11/system Xc− was not directly assessed, so this should not be

Study	Primary research?	Glioma/GBM model?	TMZ include d?	Pharmacological xCT/system Xc−-relevant intervention?	xCT/SLC7A11 directly measured or manipulated?	Eligible as core evidence?	Reason
							treated as xCT-specific evidence.
Riedel et al. 2016	Yes	Yes: six GBM cell lines: U87MG, DKMG, CAS-1, U251, BS153 and LN229	Yes	Sorafenib combined with TMZ	No	Borderline/supportive negative multi-target evidence	Direct sorafenib+TMZ testing using clonogenic survival, but no xCT/SLC7A11 mechanism. Sorafenib inhibited proliferation but failed to enhance TMZ-induced cell inactivation and showed a trend towards resistance in some cell lines. This is important negative sorafenib/TMZ evidence and prevents overclaiming.
Jakubowicz-Gil et al. 2017	Yes	Yes: T98G glioblastoma and MOGGCCM anaplastic astrocytoma cells	Yes	Sorafenib combined with TMZ	No	Borderline/supportive programmed-cell-death evidence	Direct TMZ+sorafenib testing in human glioma cells. The study assessed programmed cell death, apoptosis, autophagy, ER stress and heat shock proteins. It supports a possible sorafenib/TMZ interaction through apoptosis/autophagy/ER-stress pathways, but does not assess xCT/SLC7A11 or ferroptosis, so it should not be treated as xCT-specific evidence.
Zhang et al. 2023	Yes	Yes: U87MG glioma cells and U87MG tumour-bearing mice	Yes	Sorafenib co-loaded with TMZ in LAT1-targeted nanoparticles	No	Borderline/supportive delivery-system evidence	Direct TMZ+sorafenib co-delivery study with in vitro and in vivo outcomes. LAT1-targeted nanoparticles improved uptake, cytotoxicity, tumour accumulation and anti-tumour efficacy. The study includes redox/ferroptosis-associated markers such as ROS, GSH and GPX4,

Study	Primary research?	Glioma/GBM model?	TMZ included?	Pharmacological xCT/system Xc−-relevant intervention?	xCT/SLC7A11 directly measured or manipulated?	Eligible as core evidence?	Reason
							but the mechanism centres on LAT1-targeted delivery and sorafenib/GPX4-related effects rather than direct xCT/system Xc− inhibition.
Wang et al. 2023	Yes	Yes: TMZ-resistant GBM models, including U251TR, LN229TR and GL261TR; postoperative/resection model	Yes	Erastin co-delivered with TMZ in cRGD-liposome/GelMA hydrogel platform	Yes: xCT was assessed alongside ferroptosis-related markers	Core/partly direct platform-based erastin/TMZ evidence	Eligible as direct TMZ+erastin evidence in TMZ-resistant GBM models with xCT, GPX4, GSH, MDA, ROS and mitochondrial ferroptosis-related findings. However, it should be interpreted as platform-based evidence because sustained release, cRGD targeting and hydrogel-liposome delivery may contribute to the observed effect. It should not be treated as proof that free erastin alone would produce the same effect.
Yi et al. 2025	Yes	Yes: GL261/TR TMZ-resistant glioma cells, bEnd.3 BBB model and orthotopic GL261/TR TMZ-resistant GBM mice	Yes	Sulfasalazine/SSZ co-delivered with TMZ in PCB-modified liposomes	Yes: x-CT expression measured; GSH, GPX4, ROS, LPO, Fe2+, DMT1, beta-catenin and MGMT also assessed	Core/partly direct platform-based TMZ/SSZ evidence	Eligible as direct TMZ+SSZ evidence in TMZ-resistant GBM models. The study reported x-CT suppression, GSH/GPX4 depletion, ROS/LPO and Fe2+ increase, MGMT downregulation, reduced tumour growth and improved survival in an orthotopic model. However, the findings should be interpreted as evidence for a targeted PCB-modified co-delivery platform, not as free TMZ+SSZ evidence alone.

Abbreviations: DFO, deferoxamine; Fer-1, ferrostatin-1; GBM, glioblastoma; GPX4, glutathione peroxidase 4; GSH, glutathione; LAT1, L-type amino acid transporter 1; LPO, lipid peroxidation; MGMT, O6-methylguanine-DNA methyltransferase; PCB, poly(carboxybetaine); ROS, reactive oxygen species; SAS/SSZ, sulfasalazine; TMZ, temozolomide; xCT/SLC7A11, light-chain subunit of system Xc−. **Note:** “Core”, “supportive” and “borderline” classifications refer to relevance to the focused review question, not to study quality.
