# Supplementary material for: Pharmacological targeting of xCT/system Xc− to modulate temozolomide sensitivity in glioma and glioblastoma preclinical models: a focused systematic review
Source: Cancer Chemother Pharmacol. 2026 Oct 9;96(1):116. doi: 10.1007/s00280-026-04957-y (PMC13652864; doi:10.1007/s00280-026-04957-y)
Supplement: Supplementary file 3 [file 280_2026_4957_MOESM3_ESM.pdf]

Supplementary Table 3. Full-text reports excluded with reasons.

Author/year	Full article title	Record type	Primary reason for full-text exclusion	Background use, if any
**Chen et al. 2015; retracted 2026**	*Erastin sensitizes glioblastoma cells to temozolomide by restraining xCT and cystathionine-γ-lyase function*	Retracted preclinical article	The article was retracted in 2026 because of concerns regarding the reliability of the reported data. It was therefore excluded from the reliable evidence synthesis.	Retained only to document the screening history and subsequent retraction. It was not used to support the review findings or conclusions.
**Robe et al. 2006**	*A phase 1–2, prospective, double blind, randomized study of the safety and efficacy of Sulfasalazine for the treatment of progressing malignant gliomas: study protocol of [ISRCTN45828668]*	Clinical trial protocol	Clinical protocol involving patients with progressing malignant glioma; it did not provide eligible preclinical evidence testing TMZ combined with pharmacological xCT/system Xc− inhibition.	Used only as background evidence of early clinical interest in sulfasalazine for malignant glioma.
**Mastall et al. 2024**	*A phase Ib/II randomized, open-label drug repurposing trial of glutamate signaling inhibitors in combination with chemoradiotherapy in patients with newly diagnosed glioblastoma: the GLUGLIO trial protocol*	Ongoing clinical trial protocol	Clinical trial protocol without completed preclinical experimental evidence answering the review question.	Used only to describe continuing translational interest in glutamate-related interventions combined with standard chemoradiotherapy.

**Takeuchi et al. 2014**	*Sulfasalazine and temozolomide with radiation therapy for newly diagnosed glioblastoma*	Clinical comparative study	Human clinical study; it did not use a preclinical glioma/glioblastoma model and therefore did not meet the review's preclinical eligibility criteria.	Used as clinical tolerability and translational context, including the reported adverse events and absence of a clear survival benefit.
**Hottinger et al. 2014**	*Phase I study of sorafenib combined with radiation therapy and temozolomide as first-line treatment of high-grade glioma*	Phase I clinical study	Human clinical study of sorafenib, radiotherapy and TMZ; it did not provide eligible preclinical evidence of pharmacological xCT/system Xc− targeting or direct xCT target assessment.	Used only as clinical safety and translational context for sorafenib-based combinations.
**de Souza et al. 2022**	*High levels of NRF2 sensitize temozolomide-resistant glioblastoma cells to ferroptosis via ABCC1/MRP1 upregulation*	Broader preclinical ferroptosis/TMZ-resistance study	The study examined TMZ resistance and vulnerability to erastin- or RSL3-induced ferroptosis, but did not directly test TMZ combined with a pharmacological xCT/system Xc− inhibitor as the intervention of interest.	Not used in the manuscript; listed only to document the full-text screening and exclusion decision..
**Su et al. 2022**	*HIF-α activation by the prolyl hydroxylase inhibitor roxadustat suppresses chemoresistant*	Broader preclinical ferroptosis/chemoresistance study	The intervention was roxadustat in chemoresistant glioblastoma models. The study did not test TMZ cotreatment with	Not used in the manuscript; listed only to document the full-text screening and exclusion decision.

	glioblastoma growth by inducing ferroptosis		an xCT/system Xc−-relevant pharmacological inhibitor.	
**Moujalled et al. 2022**	*BH3 mimetic drugs cooperate with Temozolomide, JQ1 and inducers of ferroptosis in killing glioblastoma multiforme cells*	Broader preclinical programmed-cell-death combination study	TMZ was combined with BH3 mimetic drugs, whereas erastin and RSL3 were tested in combination with BH3 mimetics separately. The study did not directly evaluate TMZ combined with erastin, RSL3 or another pharmacological xCT/system Xc− inhibitor.	Not used in the manuscript; listed only to document the full-text screening and exclusion decision.
**Yang et al. 2022**	*Inhibitory effects of temozolomide on glioma cells is sensitized by RSL3-induced ferroptosis but negatively correlated with expression of ferritin heavy chain 1 and ferritin light chain*	Broader preclinical GPX4/ferroptosis–TMZ study	The study directly tested RSL3 with TMZ, but RSL3 inhibits GPX4 rather than xCT/system Xc−. It therefore fell outside the focused pharmacological target specified in the eligibility criteria.	Used only as background evidence concerning GPX4-directed ferroptosis and TMZ response.
