# Supplementary material for: Pharmacological targeting of xCT/system Xc− to modulate temozolomide sensitivity in glioma and glioblastoma preclinical models: a focused systematic review
Source: Cancer Chemother Pharmacol. 2026 Oct 9;96(1):116. doi: 10.1007/s00280-026-04957-y (PMC13652864; doi:10.1007/s00280-026-04957-y)
Supplement: Supplementary file 4 [file 280_2026_4957_MOESM4_ESM.pdf]

Supplementary Table 4. Study-level quality, reporting and applicability assessment

Study	Model identity/ relevance	Concentration and duration reported	Single-agent and combination controls	Outcome method appropriate	Statistical analysis reported	Reproducibility/ replicates reported	Randomisation	Blinding	Direct functional xCT assessment	Ferroptosis rescue/ validation	In-vivo relevance	Study limitations acknowledged	Support for judgement
**Ignarro et al. 2016**	Y	Y	Y	Y	Y	Y	NA	NA	N	N	N	Y	A172 and T98G human GBM cells were treated with 25 μM TMZ and/or 0.5 mM SAS for 1, 3 or 5 days. Vehicle, single-agent and combination groups were included. MTT viability, transcriptomic, GSH and PCNA outcomes were assessed. Viability experiments were performed independently four times in triplicate; GSH and PCNA experiments also reported independent repeats. No cystine transport, glutamate-release or other functional xCT assay was performed, and ferroptosis was not tested using rescue or specific validation. The authors acknowledged uncertainty concerning the clinically corresponding in-vitro SAS concentration.
**Sehm et al. 2016a**	Y	Y	Y	Y	Y	Y	NA	NA	Y	Y	N	Y	F98 and U251 cells with xCT overexpression or knockdown were tested with TMZ, SAS, erastin and sorafenib using reported concentrations and durations. Controls and replicate/sample numbers were reported. xCT relevance was examined through genetic manipulation and extracellular glutamate release. DFO and ferrostatin-1 rescued erastin-induced death, supporting ferroptosis, although this did not establish that TMZ-induced death itself was ferroptotic. The authors acknowledged inhibitor non-specificity, species/model differences and the absence of tumour-microenvironment assessment.
**Polewski et al. 2016**	Y	Y	Y	Y	Y	Y	NA	NA	Y	N	N	Y	U251, U87, PBT017 and normal astrocyte models were used, including stable SLC7A11 overexpression and knockdown. TMZ/SSZ dose-response experiments and appropriate untreated, single-treatment and combination groups were included. Experiments were performed in at least triplicate and repeated on at least three independent occasions. Functional relevance was supported by SLC7A11 manipulation, glutamate release, GSH and ROS measurements. No ferroptosis-rescue experiment was conducted. Orthotopic samples were used to examine xCT expression, but no in-vivo TMZ/SSZ efficacy experiment was performed. The authors acknowledged potential SSZ off-target effects, incomplete knockdown and unresolved signalling mechanisms.
**Sehm et al. 2016b**	Y	Y	Y	Y	Y	Y	N	N	Y	Y	Y	Y	F98 and U251 cells, primary astrocytes and neurons, organotypic brain tissue and an orthotopic glioma model were used. SAS and TMZ exposures, single-agent and combination comparisons and replicate numbers were reported. Extracellular glutamate release directly assessed xCT transport function. DFO and ferrostatin-1 rescued high-concentration SAS-induced death. The in-vivo component assessed SAS effects on tumour growth and glioma-associated oedema, but did not test TMZ+SAS. Animal randomisation and blinding were not reported. The authors acknowledged the high SAS concentrations required for cytotoxicity and the need for more potent or clinically feasible compounds.
**Wei et al. 2021**	Y	Y	Y	Y	Y	Y	Y	N	N	N	Y	Y	U251, SHG-44, C6 and LN18 glioma cells and a subcutaneous U251 xenograft model were used. TMZ and sorafenib concentrations, exposure periods, vehicle, single-agent and combination groups were reported. MTT, apoptosis, mitochondrial, ROS, autophagy and JAK2/STAT3-AIF outcomes were assessed, and experiments were repeated independently. Mice were randomly allocated to treatment groups, but blinding was not reported. xCT/SLC7A11 was not assessed, and the study investigated oxidative stress, autophagy and apoptosis rather than ferroptosis. The authors acknowledged that an intracranial model was not used and that orthotopic validation remained necessary.
**Riedel et al. 2016**	Y	Y	Y	Y	Y	Y	NA	NA	N	N	N	Y	Six MGMT-negative human GBM cell lines were tested using reported sorafenib and TMZ exposures. Vehicle, single-agent and combination groups were assessed. Clonogenic survival was an appropriate method for determining durable reproductive cell survival, supported by proliferation and signalling assays. Experiments were performed repeatedly with replicate cultures. No xCT/system Xc− or ferroptosis assessment was conducted, and there was no animal experiment. The authors acknowledged that in-vitro clonogenic assays might not capture effects on tumour stem cells, angiogenesis or the tumour microenvironment.
**Jakubowicz-Gil et al. 2017**	Y	Y	Y	Y	Y	Y	NA	NA	N	N	N	Y	T98G GBM and MOGGCCM anaplastic astrocytoma cells were exposed to sorafenib, TMZ or both for 48 hours. Control, single-agent and combination groups were included. Microscopy, flow cytometry, mitochondrial membrane potential, immunoblotting, caspase assays and siRNA experiments were used. Experiments were repeated three times, with at least 1,000 cells evaluated in microscopy experiments. No xCT or ferroptosis-specific assessment was performed and no in-vivo model was used. The authors discussed discrepancies between microscopy and flow-cytometry estimates, cell-line-specific responses and limitations in attributing the observed death exclusively to particular pathways.
**Zhang et al. 2023**	Y	Y	Y	Y	Y	Y	Y	N	N	N	Y	N	U87MG cells, a BBB-related delivery assessment and U87MG tumour-bearing mice were used. TMZ and sorafenib loading, in-vitro exposure and in-vivo dosing were reported. Controls included PBS, individual drug-loaded nanoparticles, non-targeted dual-drug nanoparticles and LAT1-targeted dual-drug nanoparticles. CCK-8, apoptosis, ROS, GSH, GPX4, biodistribution, tumour-growth and survival outcomes were assessed, with group sizes reported. Mice were randomly allocated, but blinding was not reported. xCT was not measured. ROS elevation, GSH depletion and GPX4 reduction were compatible with ferroptosis but were not confirmed by rescue, lipid-peroxidation, iron-dependence or equivalent specific validation. No explicit study-specific limitations were acknowledged.
**Wang et al. 2023**	Y	Y	Y	Y	Y	Y	Y	N	N	Y	Y	Y	TMZ-resistant U251TR, LN229TR and GL261TR models and a postoperative orthotopic resection model were used. Free-drug interaction experiments, individual agents, single-loaded formulations and combined hydrogel-liposome formulations were compared. Concentrations, ratios, exposure periods, release profiles and animal doses were reported. Replicate numbers and animal group sizes were stated, and animals were randomly assigned; blinding was not reported. xCT expression was measured, but functional transport or causal xCT target engagement was not established. Ferroptosis was supported by multiple measures, including GPX4, ferritin, GSH, MDA, ROS and ferroptosis-compatible mitochondrial changes, although no rescue experiment was conducted for the combination. The authors acknowledged erastin's poor solubility and that proposed mechanistic findings required further investigation.
**Yi et al. 2025**	Y	Y	Y	Y	Y	Y	Y	N	N	Y	Y	N	GL261/TR TMZ-resistant cells, a bEnd.3 BBB model and orthotopic GL261/TR tumours were used. Free TMZ, free SSZ, free combination and unmodified, PEG-modified and PCB-modified co-loaded liposome groups were compared. Concentrations, exposure periods, pharmacokinetic sampling and animal doses were reported. Replicate and animal group sizes were stated, and mice were randomly allocated; blinding was not reported. xCT expression was measured, but cystine transport, glutamate exchange or causal xCT manipulation was not performed. Ferroptosis was supported by xCT/GPX4 changes, GSH depletion, ROS, C11-BODIPY lipid peroxidation, Fe^{2+} accumulation, DMT1 and tissue iron assessment, although no ferroptosis-rescue experiment was reported. No explicit study-specific limitations were acknowledged.

Note: Y, yes or clearly reported; N, no, not performed or not reported; U, unclear; NA, not applicable. This matrix summarises study-level reporting, internal-validity indicators and applicability to the review question. It was used to guide interpretation of the preclinical evidence and was not intended to replace a clinical risk-of-bias tool. For the "Study limitations acknowledged" domain, Y indicates that the authors explicitly discussed at least one study-specific methodological, interpretive or translational limitation, even if the paper did not contain a separate limitations heading. For "In-vivo relevance", descriptive use of tumour tissue without an in-vivo treatment experiment relevant to the intervention was not counted as in-vivo efficacy evidence.
