# Supplementary material for: Pharmacological targeting of xCT/system Xc− to modulate temozolomide sensitivity in glioma and glioblastoma preclinical models: a focused systematic review
Source: Cancer Chemother Pharmacol. 2026 Oct 9;96(1):116. doi: 10.1007/s00280-026-04957-y (PMC13652864; doi:10.1007/s00280-026-04957-y)
Supplement: Supplementary file 5 [file 280_2026_4957_MOESM5_ESM.pdf]

PRISMA 2020 Checklist

Manuscript: Pharmacological targeting of xCT/system Xc− to modulate temozolomide sensitivity in glioma and glioblastoma preclinical models: a focused systematic review

PRISMA reporting statement: This focused systematic review was reported in accordance with the PRISMA 2020 statement where applicable. The review used narrative synthesis and did not include a meta-analysis; items requiring statistical synthesis, formal sensitivity analysis, formal reporting-bias assessment or formal certainty grading are therefore identified as not applicable or not formally assessed, as appropriate.

How to use this checklist: Locations refer to the section names and page numbers in the current 22-page manuscript. Page numbers should be checked again if the manuscript is reformatted before submission. Supplementary-table titles are given where they provide the principal supporting information. Rows shaded pale yellow identify PRISMA details that are not fully stated in the current manuscript and should not be interpreted as completed reporting.

Section and topic	Item	PRISMA 2020 checklist item	Location in manuscript	Reporting status / notes
**Title**	1	Identify the report as a systematic review.	Title, p. 1.	Reported. The title identifies the article as a focused systematic review.
**Abstract**	2	See the PRISMA 2020 for Abstracts checklist.	Abstract, pp. 1–2.	Reported in a structured abstract with Background, Methods, Results and Conclusions. It states the review objective, sources, eligibility, narrative synthesis, ten included studies, principal limitations and cautious implications.
**Rationale**	3	Describe the rationale for the review in the context of existing knowledge.	Background, pp. 2–4.	Reported. The rationale links TMZ resistance with redox regulation, glutathione synthesis, xCT/system Xc− biology and ferroptosis-related vulnerability, while distinguishing SAS/SSZ, erastin and multi-target sorafenib.
**Objectives**	4	Provide an explicit statement of the objective(s) or question(s) the review addresses.	End of Background, p. 4; Review design and registration, p. 4.	Reported. The review evaluates whether pharmacological xCT/system Xc− inhibition enhances TMZ sensitivity in glioma/glioblastoma preclinical models.
**Eligibility criteria**	5	Specify the inclusion and exclusion criteria for the review and how studies were grouped for the syntheses.	Eligibility criteria, pp. 4–5; Quality assessment and synthesis, pp. 5–6; Supplementary Table 2.	Reported. Criteria cover primary preclinical glioma/GBM models, TMZ cotreatment, xCT/system Xc−-relevant pharmacological interventions and relevant outcomes. Studies were synthesised by inhibitor type, evidence format and directness of xCT/system Xc− assessment.
**Information sources**	6	Specify all databases, registers, websites, organisations, reference lists and other sources searched or consulted to identify studies. Specify the date when each source was last searched or consulted.	Search strategy, p. 4; Supplementary Table 1.	Reported. Web of Science, PubMed/MEDLINE, the Cochrane Library, supplementary Google Scholar screening and citation/reference checking are identified. Searches were conducted from October 2025 to April 2026 and completed on 30 April 2026.

**Search strategy**	7	Present the full search strategies for all databases, registers and websites, including any filters and limits used.	Search strategy, p. 4; Supplementary Table 1.	Reported. Source-specific search strings are provided, no lower publication-date restriction was applied, and the first 60 Google Scholar results were screened manually. Platform/interface, searched fields and any additional source-specific limits should be interpreted from the final Supplementary Table 1.
**Selection process**	8	Specify the methods used to decide whether a study met the inclusion criteria of the review, including how many reviewers screened each record and each report retrieved, whether they worked independently, and if applicable, details of automation tools used in the process.	Study selection and data extraction, p. 5.	Reported. All five authors independently searched the selected databases; titles/abstracts were screened against predefined criteria; full texts were assessed where eligibility was uncertain; potentially eligible studies were discussed by the full team; disagreements were resolved by consensus.
**Data collection process**	9	Specify the methods used to collect data from reports, including how many reviewers collected data from each report, whether they worked independently, any processes for obtaining or confirming data from study investigators, and if applicable, details of automation tools used in the process.	Study selection and data extraction, p. 5.	Reported. Data extraction was performed by the review team and eligibility decisions, extracted data and study classifications were checked by the authors during manuscript preparation.
**Data items**	10a	List and define all outcomes for which data were sought. Specify whether all results that were compatible with each outcome domain in each study were sought, and if not, the methods used to decide which results to collect.	Eligibility criteria and Study selection and data extraction, pp. 4–5.	Reported. Outcomes sought included TMZ sensitivity, viability, cytotoxicity, apoptosis/cell death, ROS, GSH, lipid peroxidation, GPX4, xCT/SLC7A11 expression, tumour growth and survival, with additional mechanistic outcomes extracted where reported.
**Data items**	10b	List and define all other variables for which data were sought. Describe any assumptions made about any missing or unclear information.	Study selection and data extraction, p. 5; Table 1; Supplementary Table 2.	Reported. Variables included publication details, cell/animal models, inhibitor and TMZ concentrations, treatment duration, comparators, formulation/delivery approach, gene expression, metabolic findings and study limitations. No imputation of missing information is reported; unclear reporting was handled cautiously in the quality matrix.
**Study risk of bias assessment**	11	Specify the methods used to assess risk of bias in the included studies, including details of the tool(s) used, how many reviewers assessed each study and whether they worked independently, and if applicable, details of automation tools used in the process.	Quality assessment and synthesis, pp. 5–6; Supplementary Table 4.	Reported. An author-developed structured checklist informed by preclinical, in-vitro and animal-study reporting domains assessed model relevance, exposure reporting, controls, outcomes, statistics, reproducibility, randomisation, blinding, xCT assessment, ferroptosis validation, in-vivo relevance and acknowledged limitations. Ratings were Y/N//NA with support for judgement.
**Effect measures**	12	Specify for each outcome the effect measure(s) used in the synthesis or presentation of results.	Study selection and data extraction, p. 5; Results, pp. 10–12; Table 1.	Reported as study-specific measures rather than a common pooled effect. Measures included viability/cytotoxicity, reported synergy or combination indices, apoptosis/cell-death outcomes, redox and ferroptosis-related marker changes, tumour growth and survival. Results were presented as reported by the original studies.

**Synthesis methods**	13a	Describe the processes used to decide which studies were eligible for each synthesis.	Eligibility criteria, pp. 4–5; Quality assessment and synthesis, pp. 5–6; Supplementary Table 2.	Reported. Eligibility was checked against the focused TMZ plus pharmacological xCT/system Xc− question. Genetic evidence was treated as supportive mechanistic evidence, sorafenib as indirect/multi-target evidence unless direct xCT assessment was available, and delivery studies as formulation-specific evidence.
**Synthesis methods**	13b	Describe any methods required to prepare the data for presentation or synthesis, such as handling of missing summary statistics, or data conversions.	Quality assessment and synthesis, pp. 5–6; Results, pp. 7–12.	Reported. Data were organised by inhibitor class, evidence format and mechanistic directness. No data conversion, imputation of missing statistics or reconstruction of common effect estimates was undertaken; findings were presented narratively as reported.
**Synthesis methods**	13c	Describe any methods used to tabulate or visually display results of individual studies and syntheses.	Figure 1, pp. 8–9; Table 1, p. 9; Supplementary Tables 2–4.	Reported. Figure 1 displays study selection; Table 1 summarises models, interventions, evidence directness, findings and interpretive limitations; Supplementary Table 2 documents eligibility; Supplementary Table 3 documents full-text exclusions; Supplementary Table 4 presents study-level quality, reporting and applicability judgements.
**Synthesis methods**	13d	Describe any methods used to synthesize results and provide a rationale for the choice(s). If meta-analysis was performed, describe the model(s), methods used to identify the presence and extent of statistical heterogeneity, and software package(s) used.	Quality assessment and synthesis, pp. 5–6.	Reported. Narrative synthesis was selected because studies differed substantially in models, inhibitors, concentrations, treatment duration, formulations, routes and outcomes. No meta-analysis or synthesis software was used.
**Synthesis methods**	13e	Describe any methods used to explore possible causes of heterogeneity among study results.	Quality assessment and synthesis, pp. 5–6; Results, pp. 10–12; Discussion, pp. 12–16.	Reported narratively. Differences were explored by inhibitor, model, concentration, duration, assay, formulation/delivery platform, in-vivo context and directness of xCT/system Xc− assessment.
**Synthesis methods**	13f	Describe any sensitivity analyses conducted to assess robustness of the synthesized results.	Not applicable.	No formal sensitivity analysis was conducted because no quantitative synthesis was performed.
**Reporting bias assessment**	14	Describe any methods used to assess risk of bias due to missing results in a synthesis (arising from reporting biases).	Not formally assessed; Strengths and limitations, pp. 16–17.	No formal reporting-bias assessment was conducted. The small, heterogeneous preclinical evidence base and lack of meta-analysis precluded formal assessment; incomplete reporting in individual studies was considered through the study-level matrix and cautious narrative interpretation.
**Certainty assessment**	15	Describe any methods used to assess certainty (or confidence) in the body of evidence for an outcome.	Quality assessment and synthesis, pp. 5–6; Strengths and limitations, pp. 16–17; Supplementary Table 4.	No formal GRADE-style certainty assessment was conducted. Confidence was interpreted narratively according to mechanistic directness, model relevance, exposure feasibility, outcome method, reproducibility, randomisation/blinding, ferroptosis validation and formulation dependence.

**Study selection**	16a	Describe the results of the search and selection process, from the number of records identified in the search to the number of studies included in the review, ideally using a flow diagram.	Results—Study selection, pp. 6–9; Figure 1, pp. 8–9.	Reported. A total of 191 records were identified (77 Web of Science, 51 PubMed/MEDLINE, 3 Cochrane Library and 60 Google Scholar); 23 duplicates were removed; 168 records were screened; 149 were excluded; 19 reports were assessed; 9 were excluded; and 10 non-retracted preclinical studies were included.
**Study selection**	16b	Cite studies that might appear to meet the inclusion criteria, but which were excluded, and explain why they were excluded.	Results—Study selection and Redox/ferroptosis-related outcomes, pp. 6–12; Supplementary Table 3.	Reported. Supplementary Table 3 lists nine individual full-text exclusions with article titles, record type, primary exclusion reason and any background use: one retracted article, four clinical/protocol/translational reports and four broader ferroptosis/TMZ-resistance reports.
**Study characteristics**	17	Cite each included study and present its characteristics.	Results—Study selection, p. 7; Table 1, p. 9; Supplementary Table 2.	Reported. All ten included studies are cited and their models, exposures, directness of xCT/system Xc− evidence, principal findings, eligibility classification and interpretive limitations are presented.
**Risk of bias in studies**	18	Present assessments of risk of bias for each included study.	Results—Quality and risk-of-bias considerations, pp. 7–8; Supplementary Table 4.	Reported. Supplementary Table 4 presents study-level Y/N/U/NA judgements and support for judgement across model relevance, exposure reporting, controls, outcome methods, statistics, reproducibility, randomisation, blinding, functional xCT assessment, ferroptosis validation, in-vivo relevance and acknowledged limitations. No composite numerical score was used.
**Results of individual studies**	19	For all outcomes, present, for each study: summary statistics for each group where appropriate and an effect estimate and its precision, ideally using structured tables or plots.	Table 1, p. 9; inhibitor-specific Results, pp. 10–12; Supplementary Table 2.	Reported narratively using the data available from each study. Concentrations, combination findings, combination indices where reported, cell-death/redox outcomes, tumour responses and survival findings are summarised. A common effect estimate and precision measure were not available because outcomes and designs were heterogeneous.
**Results of syntheses**	20a	For each synthesis, briefly summarise the characteristics and risk of bias among contributing studies.	Results—Quality and risk-of-bias considerations, pp. 7–8; inhibitor-specific Results, pp. 10–12; Table 1; Supplementary Table 4.	Reported. The SAS/SSZ, erastin and sorafenib syntheses describe contributing models, concentrations, evidence directness and limitations. Direct mechanistic evidence, indirect multi-target evidence, negative evidence and formulation-specific evidence are distinguished.
**Results of syntheses**	20b	Present results of all statistical syntheses conducted. If meta-analysis was done, present the summary estimate, its precision and measures of statistical heterogeneity.	Not applicable.	No statistical synthesis or meta-analysis was conducted.
**Results of syntheses**	20c	Present results of all investigations of possible causes of heterogeneity among study results.	Results, pp. 10–12; Discussion, pp. 12–16.	Reported narratively. Heterogeneity is linked to cell line/species, xCT status, drug concentration, treatment duration, short-term metabolic versus clonogenic outcomes, free-drug versus platform delivery, BBB/local delivery and in-vivo model type.

**Results of syntheses**	20d	Present results of all sensitivity analyses conducted to assess the robustness of the synthesized results.	Not applicable.	No formal sensitivity analysis was conducted.
**Reporting biases**	21	Present assessments of risk of bias due to missing results (arising from reporting biases) for each synthesis assessed.	Not formally assessed; Strengths and limitations, pp. 16–17.	No formal reporting-bias assessment was performed or presented. The review instead notes incomplete methodological reporting and the limited, heterogeneous evidence base as constraints on interpretation.
**Certainty of evidence**	22	Present assessments of certainty (or confidence) in the body of evidence for each outcome assessed.	Results—Quality and risk-of-bias considerations, pp. 7–8; Discussion and Strengths and limitations, pp. 12–17; Supplementary Table 4.	No formal certainty grade was assigned. Confidence is expressed narratively: strongest for genetic/functional xCT evidence in selected studies; lower for studies without direct target assessment; preliminary and formulation-specific for delivery-platform studies; and mixed/non-specific for sorafenib.
**Discussion**	23a	Provide a general interpretation of the results in the context of other evidence.	Discussion, pp. 12–16.	Reported. Findings are interpreted in relation to xCT/system Xc− biology, redox regulation, ferroptosis-compatible mechanisms, concentration dependence, delivery effects and available clinical/translational context.
**Discussion**	23b	Discuss any limitations of the evidence included in the review.	Strengths and limitations, pp. 16–17; Table 1; Supplementary Table 4.	Reported. Limitations include only ten studies, a smaller direct free-drug evidence base, established cell lines, model and exposure heterogeneity, metabolic assays, incomplete functional target assessment, inconsistent ferroptosis rescue, small animal groups, limited blinding, uncertain human exposure and platform-specific effects.
**Discussion**	23c	Discuss any limitations of the review processes used.	Strengths and limitations, pp. 16–17; Search strategy, p. 4.	Reported. The review was not prospectively registered; Google Scholar was supplementary; citation checking was not recorded numerically; complete automated exports and duplicate records were unavailable for every source; some counts were reconstructed; and the focused criteria excluded broader non-xCT ferroptosis/TMZ studies.
**Discussion**	23d	Discuss implications of the results for practice, policy, and future research.	Discussion, pp. 12–16; Conclusions, p. 17.	Reported. The manuscript does not support a clinical recommendation. It calls for clinically justified exposures, direct target engagement, ferroptosis validation, patient-derived/TMZ-resistant and orthotopic models, pharmacokinetic/BBB evaluation, toxicity assessment and evaluation of delivery-platform safety and feasibility.
**Registration and protocol**	24a	Provide registration information for the review, including register name and registration number, or state that the review was not registered.	Review design and registration, p. 4.	Reported. The review was not prospectively registered and no registration number is available.
**Registration and protocol**	24b	Indicate where the review protocol can be accessed, or state that a protocol was not prepared.	Review design and registration, p. 4.	The manuscript was not prospectively registered.

**Registration and protocol**	24c	Describe and explain any amendments to information provided at registration or in the protocol.	Not applicable.	No registration or accessible protocol was reported; therefore, registered/protocol amendments are not applicable.
**Support**	25	Describe sources of financial or non-financial support for the review, and the role of the funders or sponsors in the review.	Declarations—Funding Sources, p. 18.	Reported. No external funding was received; consequently, there was no funder or sponsor role in the review.
**Competing interests**	26	Declare any competing interests of review authors.	Declarations—Conflict of Interest Statement, p. 18.	Reported. The authors declare no conflicts of interest related to the review.
**Availability of data, code and other materials**	27	Report which data, code and other materials are publicly available and where they can be found.	Declarations—Availability of data and materials, p. 19; Supplementary Tables 1–4.	Reported. The manuscript states that all data were extracted from published studies. Supplementary materials provide the search strategies, included-study eligibility assessment, full-text exclusions and study-level quality/reporting/applicability matrix. No analytic code was used for meta-analysis, and no public repository for screening/extraction records is identified.
